# Appraising the performance of genotyping tools in the prediction of coreceptor tropism in HIV-1 subtype C viruses

**DOI:** 10.1186/1471-2334-12-203

**Published:** 2012-09-02

**Authors:** Saleema Crous, Ram Krishna Shrestha, Simon A Travers

**Affiliations:** 1 South African National Bioinformatics Institute, University of the Western Cape, Private Bag X17, Belville, 7535, South Africa

**Keywords:** Human immunodeficiency virus, Coreceptor, Chemokine receptors, CXCR4, CCR5, Genotype, Phenotype, Subtype C

## Abstract

**Background:**

In human immunodeficiency virus type 1 (HIV-1) infection, transmitted viruses generally use the CCR5 chemokine receptor as a coreceptor for host cell entry. In more than 50% of subtype B infections, a switch in coreceptor tropism from CCR5- to CXCR4-use occurs during disease progression. Phenotypic or genotypic approaches can be used to test for the presence of CXCR4-using viral variants in an individual’s viral population that would result in resistance to treatment with CCR5-antagonists. While genotyping approaches for coreceptor-tropism prediction in subtype B are well established and verified, they are less so for subtype C.

**Methods:**

Here, using a dataset comprising V3 loop sequences from 349 CCR5-using and 56 CXCR4-using HIV-1 subtype C viruses we perform a comparative analysis of the predictive ability of 11 genotypic algorithms in their prediction of coreceptor tropism in subtype C. We calculate the sensitivity and specificity of each of the approaches as well as determining their overall accuracy. By separating the CXCR4-using viruses into CXCR4-exclusive (25 sequences) and dual-tropic (31 sequences) we evaluate the effect of the possible conflicting signal from dual-tropic viruses on the ability of a of the approaches to correctly predict coreceptor phenotype.

**Results:**

We determined that geno2pheno with a false positive rate of 5% is the best approach for predicting CXCR4-usage in subtype C sequences with an accuracy of 94% (89% sensitivity and 99% specificity). Contrary to what has been reported for subtype B, the optimal approaches for prediction of CXCR4-usage in sequence from viruses that use CXCR4 exclusively, also perform best at predicting CXCR4-use in dual-tropic viral variants.

**Conclusions:**

The accuracy of genotyping approaches at correctly predicting the coreceptor usage of V3 sequences from subtype C viruses is very high. We suggest that genotyping approaches can be used to test for coreceptor tropism in HIV-1 group M subtype C with a high degree of confidence that they will identify CXCR4-usage in both CXCR4-exclusive and dual tropic variants.

## Background

To enable cell entry by HIV, the gp120 glycoprotein, present in a trimeric arrangement on the surface of a HIV virion, must first bind to a CD4 receptor on the target cell [1-3]. This binding induces a conformational change in the gp120/gp41 trimer complex [4,5] thereby enabling binding of a chemokine receptor, either CCR5 or CXCR4 [6]. CCR5-tropic viruses are associated with primary transmission and can persist throughout infection [6]. In as many as 50% of HIV-1 subtype B infections, a switch to CXCR4-usage has been observed and this switch is generally regarded as an indicator of disease progression [7-10]. Early studies of HIV-1 subtype C suggested that a switch to CXCR4-usage was less common in subtype C compared to subtype B [11,12], however more recent studies have suggested that between 30-50% of subtype C infected individuals exhibit a change to CXCR4-usage during disease progression [13-18].

Dual-tropic viruses (R5X4) capable of using either CCR5 or CXCR4 for host cell entry have been described [19] as have dual-tropic viruses that, while capable of using either receptor for cell entry, exhibit preferential use of either CCR5 (dual-R) or CXCR4 (dual-X) [20,21]. Detecting the presence of dual-tropic viruses in an individual’s viral population is difficult however, as a mixed population of R5 and X4 viruses will be identified as dual in a population-based phenotyping assay.

Determining the coreceptor usage profile of an individual’s viral population has been used as an indicator of disease progression and in more recent years as an approach for detecting resistance to CCR5 antagonists such as maraviroc [22-24]. Phenotypic assays, such as Monogram Bioscience’s Trofile™ assay [25], are the most effective means of elucidating the coreceptor tropism of a viral population. These approaches, however, are expensive, laborious and unavailable for routine use in all laboratories [26,27]. Thus, genotyping approaches have been suggested to be a viable alternative for routine coreceptor tropism testing [28]. While many amino acid positions throughout gp120 have been suggested to influence coreceptor affinity and tropism [29-35], the V3 loop appears to be the strongest determinant of coreceptor tropism with amino acid mutations affecting V3 net charge, charge at positions 11, 24 and 25 and glycan binding patterns all implicated in causing a switch from CCR5- to CXCR4-usage [36-41].

Early genotypic algorithms predicted the coreceptor tropism of HIV-1V3 sequences using the properties of the amino acids at positions 11 and 25 while later algorithms account for various properties of the entire V3 loop [39,40,42-45]. With the exception of C-PSSM [43] and the Raymond combined 11/25 and net charge rules [46], all of these approaches have been optimised for coreceptor tropism prediction in subtype B and show varying levels of sensitivity at predicting CXCR4-usage in subtype B [47].

Despite HIV-1 subtype C accounting for almost 60% of worldwide HIV infections [48], the genetic determinants of the switch in coreceptor use are less-well understood than in subtype B. Conflicting reports have been published with some suggesting that these determinants are the same for subtype C as subtype B [46], while others have presented evidence to the contrary [43]. Jensen and colleagues developed the only subtype C specific genotyping tool with a reported sensitivity of 75% [43] while others evaluated the ability of this and other algorithms trained on subtype B data at correctly predicting CXCR4-use in subtype C sequence data [46]. They found that the most appropriate approach for predicting CXCR4-usage in subtype C were C-PSSM and their combined 11/25 and net charge rule [46]. When specificity was considered, however, Raymond and colleagues approach was significantly better than C-PSSM (96.4% versus 81.8%). The dataset used in this study, however, did not represent the entire spectrum of HIV-1 subtype C diversity in that it had a limited number of phenotyped sequences (55 R5 and 15 X4 sequences) collected from only two countries (Malawi and France).

In this study we have collated a large dataset consisting of all obtainable subtype C sequences with experimentally verified coreceptor tropism and used this to evaluate the performance of various genotyping tools at accurately predicting CXCR4-usage in HIV-1 subtype C. Further, we determine the effect of sequences from dual-tropic viruses on the sensitivity of genotyping methods.

## Results and discussion

In total 731 HIV-1 group M subtype C V3 sequences with experimentally verified coreceptor tropism were retrieved. Only one representative sequence for each individual was retained reducing the total number of sequences to 405. The final analysis dataset (available on request) contained sequences from 349 CCR5-using and 56 CXCR4-using viruses. Sequences from CXCR4-using viruses were further separated into R5X4 (dual-tropic) and CXCR4-exclusive viruses with 31 and 25 sequences, respectively, comprising these datasets.

The coreceptor usage of every sequence in each of the datasets was predicted using all of the genotyping approaches. 23 of the sequences tested contained at least one ambiguous nucleotide position. Geno2pheno is the only one of the tools tested that is capable of accounting for ambiguous positions in its genotypic predictions [44]. To assess all of the other approaches, we translated the nucleotide sequences into all the possible combinations of amino acid sequences and if one or more of these translated sequences was predicted as CXCR4-using, the genotyping call for the original sequence was taken as X4. For each of the 23 sequences, all possible translations of the sequence had the same coreceptor tropism prediction for each method. Thus, in this data, ambiguous positions did not affect the genotypic predictions. However, in many cases the presence of ambiguous nucleotide calls, particularly within the codons encoding for amino acid positions 11, 24 and 25, would substantially reduce the ability of approaches to correctly predict coreceptor usage [44]. Thus, the ability to account for ambiguous nucleotide positions in geno2pheno gives it a distinct advantage over all of the other approaches tested here.

**Figure 1:**
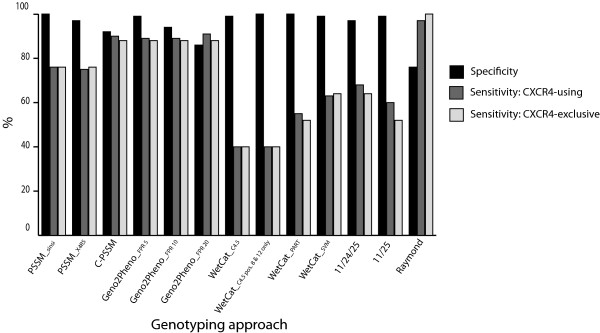
**Performance of each of the genotyping algorithms in predicting CXCR4-usage.** Sensitivity for both the CXCR4-using and CXCR4-exclusive datasets was calculated as the number of viral sequences predicted as CXCR4-using divided by the total number of CXCR4-using or CXCR4-exclusive sequences tested. Specificity corresponds to the number of CCR5-using viruses predicted as R5 divided by the total number of CCR5-using viral sequences evaluated.

The sensitivity of each of the tested approaches at predicting X4 viruses in the CXCR4-using dataset (dual tropic and CXCR4-exclusive combined) varied widely from 40-97% (Table 1 and Figure 1). The method by Raymond and colleagues performed best with 97% sensitivity while Geno2pheno (FPR20) and C-PSSM exhibited high sensitivities greater than 90%. Two variants of the wetcat package, C4.5 and C4.5 with p8-p12, performed most poorly with sensitivities of 40%, consistent with previous observations on both subtype B and non-B subtypes [46,47,49]. 

**Table 1:** Performance of genotyping approaches at predicting CXCR4-usage in viral sequences from individuals infected with HIV-1 group M subtype C

**Method**	**CXCR4-using sensitivity (%)**	**Specificity**
PSSM__sinsi_	76	100
PSSM__X4R5_	75	97
C-PSSM	90	92
Geno2Pheno__FPR5_	89	99
Geno2Pheno__FPR10_	89	94
Geno2Pheno__FPR20_	91	86
WetCat__C4.5_	40	99
WetCat__C4.5 pos. 8&12_	40	100
WetCat__PART_	55	100
WetCat__SVM_	63	99
11/24/25	68	97
11/25	60	99
Raymond	97	76

Sensitivity corresponds to the ability of the approach to predict CXCR4-use, while specificity corresponds to the ability to correctly predict CCR5-use.

While predicting CXCR4-usage with high accuracy is important, the ability to correctly identify R5 variants as CCR5-using is equally as important in reducing the amount of false positives that would result in incorrect clinical interpretations. Thus, we also calculated the specificity (proportion of CCR5-tropic viruses correctly predicted as R5) of each approach. All approaches performed well with three having 100% specificity, eight having specificity greater than 90% and geno2pheno (FPR20) and Raymond exhibiting lower specificity of 86% and 76% respectively (Table 1 and Figure 1). These high specificity values are consistent with previous observations in both HIV-1 subtype B and non-B subtypes that all approaches, in general, are better at correctly predicting CCR5-usage than CXCR4-usage [22,43,46,47,49].

Raymond and colleagues had previously evaluated nine of the 13 approaches studied here using a smaller, geographically limited subtype C dataset comprising 55 R5 and 15 X4 viral sequences sampled from Malawi and France [46]. They reported that the optimal approach for subtype C genotyping was a combination of the 11/25 and net charge rules with sensitivity and specificity for CXCR4-usage prediction in subtype C of 93.3% and 96.4% respectively. Using the larger and more geographically diverse dataset studied here, we estimate sensitivity of 97% and a specificity of 76% for this approach. Compared to the other approaches tested, however, Raymond’s method is not the optimal approach. While it does show the highest sensitivity, it also has the lowest specificity of all the approaches tested (Table 1). For the other approaches we find that sensitivity increases by as much as 22% for five of the approaches relative to the Raymond study, while the sensitivity of PSSM_sinsi_, PSSM_X4R5_ and C-PSSM drops by 4%, 5% and 3% respectively. We suggest that the weaker performance on our comprehensive subtype C dataset of the combined 11/25 and net charge rule proposed by Raymond and colleagues is most likely an artifact of the limited sample size/diversity in their dataset that is not present in the larger dataset studied here. In describing C-PSSM, Jensen and colleagues used a dataset consisting 228 R5 sequences and 51 X4 sequences (from 200 and 20 subjects respectively) [43] and reported a sensitivity of 75%, substantially less than the 90% sensitivity reported here, with comparable specificities of 94% and 92%.

While some methods are extremely sensitive at correctly predicting CXCR4-use, the optimum approach for clinical implementation also needs to be highly specific in correctly identifying viruses that do not use CXCR4. Thus, we have calculated an accuracy score for each of the approaches tested that takes into account an approach’s sensitivity and specificity (Table 2). For the CXCR4-using dataset, we find that three of the 13 approaches tested have an accuracy of 90% or greater at predicting coreceptor usage in HIV-1 group M subtype C viral sequences with geno2pheno (FPR5) being the most accurate of all approaches tested with an accuracy of 94% (89% sensitivity and 99% specificity, Table 2). Two variants of the wetcat package, C4.5 and C4.5 with p8-p12, both perform poorest with accuracy scores of 70% (Table 2).

**Table 2:** Accuracy of genotyping approaches at correctly predicting coreceptor tropism

**Method**	**CXCR4-using accuracy**	**CXCR4-exclusive accuracy**	**R5X4 accuracy**
PSSM__sinsi_	88	88	88
PSSM__X4R5_	86	87	86
C-PSSM	91	90	91
Geno2Pheno__FPR5_	94	93	94
Geno2Pheno__FPR10_	92	91	92
Geno2Pheno__FPR20_	88	87	90
WetCat__C4.5_	70	70	70
WetCat__C4.5 pos. 8&12_	70	70	70
WetCat__PART_	77	76	79
WetCat__SVM_	81	82	81
11/24/25	81	81	82
11/25	79	76	82
Raymond	86	88	85

Accuracy scores are presented for a combined dataset containing CXCR4-using viruses (both CXCR4-exclusive and dual-tropic viruses) as well as separately for the CXCR4-exclusive and dual-tropic viral sequences.

**Figure 2:**
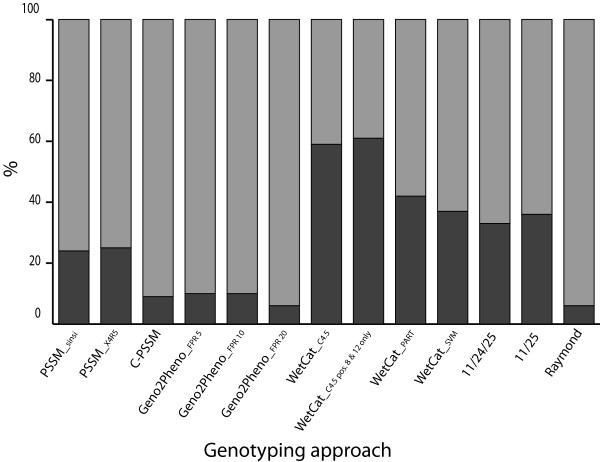
**Ability of each approach at predicting CXCR4-usage in dual-tropic viral sequences.** The percentage of dual-tropic sequences predicted as CCR5-using and CXCR4-using is shown with dark and light shaded areas of each bar corresponding to the percentage of sequences predicted as CCR5-using and CXCR4-using respectively.

Dual-tropic viruses are a unique class of viruses in that they can enter host cells using either CCR5 or CXCR4 chemokine receptors, however, some dual-tropic viruses can exhibit preferential use of one of these [19-21]. From a clinical perspective, it is imperative that genotyping approaches correctly identify the CXCR4-using capabilities of dual-tropic viruses. Genotyping algorithms have been shown to vary widely in their predictive ability of CXCR4-usage in subtype B dual-tropic viruses [50]. In general, approaches were observed to underestimate the frequency of CXCR4-usage in dual tropic viruses [50]. Thus, we sought to investigate the effect of dual-tropic viruses on the accuracy of each of the genotyping approaches tested. The CXCR4-using viruses were separated into CXCR4-exclusive and dual-tropic viral sequences and the accuracy of each of the approaches at correctly predicting coreceptor tropism was calculated (Table 2). When dual-tropic sequences are excluded, the accuracy of three of the approaches increases minimally, with four methods showing no change in accuracy and six showing a slight decrease of 1% in accuracy (Table 2). Similarly, when the dual-tropic viruses were studied separately there was minimal effect on the accuracy of each of the approaches (Table 2). There was significant variability in the ability of the approaches to accurately predict CXCR4-usage in dual-tropic viruses, ranging from 40% (wetcat C4.5 with p8-p12) to 94% (Geno2pheno FPR20) of sequences from dual-tropic viruses predicted as CXCR4-using (Figure 2). It appears that, in subtype C at least, the ability of approaches to predict CXCR4-usage in dual tropic viruses directly correlates with their ability to predict CXCR4-usage in CXCR4-exclusive viruses. Such an observation does not appear to hold true in subtype B, however, where some methods with high sensitivity for prediction of CXCR4 viruses in subtype B [47], show low accuracy for the prediction of CXCR4-usage in subtype B dual-tropic viral sequences [50]. Geno2pheno, however, does show high accuracy (90%) for the prediction of CXCR4-usage in subtype B dual-tropic viruses [50]. 

## Conclusion

Using a comprehensive, geographically diverse dataset, we find that geno2pheno (FPR5) is the most accurate approach for the prediction of coreceptor tropism in HIV-1 subtype C viral sequences. Coupled with it’s high accuracy, the ability of geno2pheno to account for ambiguous nucleotide calls in V3 sequences gives it a distinct advantage over all other approaches for coreceptor genotyping of sequence data generated from population-based sequencing. We also report that in HIV-1 group M subtype C, sequences from dual-tropic viruses have minimal effect on accuracy calculations and the optimal approaches for prediction of CXCR4-usage in sequence from viruses that use CXCR4 exclusively also perform best at predicting CXCR4-use in dual-tropic viral variants. Based on this work we suggest that viral genotyping of envelope sequences from subtype C infected individuals is feasible with the correct approach and can be undertaken with a high degree of confidence that CXCR4-usage will be accurately identified in both CXCR4-exclusive and dual tropic variants.

## Methods

### Study data

A dataset consisting of 731 HIV-1 subtype C V3 nucleotide sequences with phenotypically determined coreceptor tropism was sourced. The majority of sequences were retrieved from the HIV LANL Sequence Database (hiv.lanl.gov), with the remainder originating from published literature [43,46]. Multiple sequence alignments were produced manually using MacClade 4.08 [51]. To avoid potential bias in results, multiple samples from the same individuals were excluded with a single representative sequence randomly selected for these individuals.

### Genotypic algorithms

The coreceptor tropism of each V3 sequence was predicted using a number of genotyping methods. These comprised PSSM_X4R5_ and PSSM_SINSI_[42] as well as the subtype C PSSM tool [43], geno2pheno [44] and four variants (C4.5, C4.5 with p8-p12, PART and SVM) of the wetcat package [45]. Tropism was also predicted using the 11/25 [39] and 11/24/25 [40] rules (software implementation available from the corresponding author on request). Raymond and colleagues recently proposed a combination of the 11/25 and charge rules for prediction of CXCR4-use in subtype C sequences [46]. One of the following criteria is required for predicting CXCR4 coreceptor usage: (1) 11 R/K and/or 25K, (2) 25R and a net charge of ≥ +5, or (3) a net charge of ≥ +6 [46]. For geno2pheno, three different false positive rates (5%, 10% and 20%) were used to determine the optimal parameters for the accurate prediction of CXCR4-usage in subtype C viral sequences. Each of the geno2pheno false positive rates used is described as an individual approach throughout the paper for clarity purposes. Sequences duplicated by each of the PSSM tools (as a result of more than one optimal alignment to the reference sequence) were only considered for further analysis when genotypic predictions made by the matrix were the same for all alignment variations. The presence of ambiguous nucleotide calls in a sequence can affect the accuracy of genotyping approaches [44]. Thus, if a tested genotyping approach was not designed to account for ambiguous nucleotide positions, all possible combinations of amino acid sequences were output and a worst-case scenario approach was employed whereby if one of these translated sequences was predicted as CXCR4-using, the genotyping call for the original sequence was taken as X4.

Viral sequences were separated into three distinct categories (R5, X4 and R5X4) based upon their experimentally verified viral phenotype. Dual-tropic and CXCR4-tropic viruses were studied both separately and together (as CXCR4-using) in order to determine the affect of the conflicting signal of dual-tropic viruses on sensitivity estimates. The sensitivity of each approach for CXCR4 prediction was calculated as the number of predicted X4 viruses in the CXCR4-using dataset divided by the total number of sequences in the CXCR4-using dataset. The specificity of each approach for CXCR4 prediction was calculated as the number of predicted R5 viruses in the CCR5-using dataset divided by the total number of sequences in the CCR5-using dataset. The same method was used to calculate the sensitivity and specificity of each genotyping method on the CXCR4-exclusive and dual-tropic datasets.

Further, an overall accuracy score for each of the approaches used was calculated using:

(1) $$\frac{TP+TN}{TP+TN+FP+FN}$$

where, for the CXCR4-using dataset, TP corresponds to the number of CXCR4-using sequences predicted as CXCR4-using, TN the number of R5 sequences predicted as CCR5-using, FP the number of R5 sequences predicted as CXCR4-using and FN the number of CXCR4-using sequences predicted as CCR5-using. For the CXCR4-exclusive dataset the TP and FN values were calculated only for sequences phenotypically determined to exclusively use CXCR4. For each calculation we normalized the TP and FN values relative to the TN and FP values to account for the disproportionate number of sequences representing the positive (CXCR4-using or CXCR4-exclusive) and negative (CCR5) datasets (see Additional file 1: Table S1 for the uncorrected values).

## Abbreviations

HIV-1: Human immunodeficiency virus type 1; FPR: False positive rate; CXCR4: C-X-C chemokine receptor type 4; CCR5: C-C chemokine receptor type 5.

## Competing interests

The authors declare that they have no competing interests.

## Authors' contributions

SC collated the dataset, tested the various genotyping approaches and wrote the first version of the manuscript. RKS wrote software for the translation of ambiguous nucleotides and for the approach by Raymond and colleagues as well as collating results from a number of the approaches used. SAAT conceived the study, participated in its design and wrote the final manuscript. All authors read and approved the final manuscript.

## Pre-publication history

The pre-publication history for this paper can be accessed here:

http://www.biomedcentral.com/1471-2334/12/203/prepub

## Supplementary Material

Additional file 1 **Table S1.** Tables detailing the uncorrected numbers of true positives (CXCR4-usage correctly predicted in CXCR4-using sequences), true negatives (CCR5-usage correctly predicted in CCR5-using sequences), false positives (CXCR4-usage incorrectly predicted in CCR5-using sequences) and false negatives (CCR5-usage incorrectly predicted in CXCR4-using sequences) predicted by each of the approaches. Results are shown for (A) CXCR4-using sequences, (B) CXCR4-exclusive sequences and (C) dual-tropic sequences. Click here for file
