# Supplementary material for: Appraising the performance of genotyping tools in the prediction of coreceptor tropism in HIV-1 subtype C viruses
Source: BMC Infect Dis. 2012 Sep 2;12:203. doi: 10.1186/1471-2334-12-203 (PMC3482586; doi:10.1186/1471-2334-12-203)
Supplement: Additional file 1 — Table S1. Tables detailing the uncorrected numbers of true positives (CXCR4-usage correctly predicted in CXCR4-using sequences), true negatives (CCR5-usage correctly predicted in CCR5-using sequences), false positives (CXCR4-usage incorrectly predicted in CCR5-using sequences) and false negatives (CCR5-usage incorrectly predicted in CXCR4-using sequences) predicted by each of the approaches. Results are shown for (A) CXCR4-using sequences, (B) CXCR4-exclusive sequences and (C) dual-tropic sequences. [file 1471-2334-12-203-S1.doc]

**Supplementary Table 1:** Tables detailing the uncorrected numbers of true positives (CXCR4-usage correctly predicted in CXCR4-using sequences), true negatives (CCR5-usage correctly predicted in CCR5-using sequences), false positives (CXCR4-usage incorrectly predicted in CCR5-using sequences) and false negatives (CCR5-usage incorrectly predicted in CXCR4-using sequences) predicted by each of the approaches. Results are shown for (A) CXCR4-using sequences, (B) CXCR4-exclusive sequences and (C) dual-tropic sequences.

**A**

| **Method** | **TP** | **TN** | **FP** | **FN** |
| --- | --- | --- | --- | --- |
| Web PSSM B sinsi | 44 | 348 | 1 | 14 |
| Web PSSM B x4r5 | 43 | 338 | 10 | 14 |
| Web PSSM C | 52 | 317 | 29 | 6 |
| Geno2Pheno – FPR 5 | 50 | 344 | 5 | 6 |
| Geno2Pheno – FPR 10 | 50 | 328 | 21 | 6 |
| Geno2Pheno – FPR 20 | 51 | 299 | 50 | 5 |
| WetCat – C4.5 | 23 | 346 | 3 | 34 |
| WetCat – C4.5 pos. 8 & 12 only | 23 | 348 | 1 | 35 |
| WetCat – PART | 32 | 348 | 1 | 26 |
| WetCat – SVM | 36 | 344 | 3 | 21 |
| 11/24/25 Charge Rule | 38 | 340 | 9 | 20 |
| 11/25 Charge Rule | 34 | 347 | 2 | 24 |
| Raymond | 55 | 262 | 82 | 2 |

**B**

|  | **TP** | **TN** | **FP** | **FN** |
| --- | --- | --- | --- | --- |
| Web PSSM B sinsi | 19 | 348 | 1 | 6 |
| Web PSSM B x4r5 | 19 | 338 | 10 | 6 |
| Web PSSM C | 22 | 317 | 29 | 3 |
| Geno2Pheno – FPR 5.5 | 22 | 344 | 5 | 3 |
| Geno2Pheno – FPR 10.5 | 22 | 328 | 21 | 3 |
| Geno2Pheno – FPR 20.5 | 22 | 299 | 50 | 3 |
| WetCat – C4.5 | 10 | 346 | 3 | 15 |
| WetCat – C4.5 pos. 8 & 12 only | 10 | 348 | 1 | 15 |
| WetCat – PART | 13 | 348 | 1 | 12 |
| WetCat – SVM | 16 | 344 | 3 | 9 |
| 11/24/25 Charge Rule | 16 | 340 | 9 | 9 |
| 11/25 Charge Rule | 13 | 347 | 2 | 12 |
| Raymond | 25 | 262 | 82 | 0 |

**C**

|  | **TP** | **TN** | **FP** | **FN** |
| --- | --- | --- | --- | --- |
| Web PSSM B sinsi | 25 | 348 | 1 | 8 |
| Web PSSM B x4r5 | 24 | 338 | 10 | 8 |
| Web PSSM C | 30 | 317 | 29 | 3 |
| Geno2Pheno – FPR 5.5 | 28 | 344 | 5 | 3 |
| Geno2Pheno – FPR 10.5 | 28 | 328 | 21 | 3 |
| Geno2Pheno – FPR 20.5 | 29 | 299 | 50 | 2 |
| WetCat – C4.5 | 13 | 346 | 3 | 19 |
| WetCat – C4.5 pos. 8 & 12 only | 13 | 348 | 1 | 20 |
| WetCat – PART | 19 | 348 | 1 | 14 |
| WetCat – SVM | 20 | 344 | 3 | 12 |
| 11/24/25 Charge Rule | 22 | 340 | 9 | 11 |
| 11/25 Charge Rule | 21 | 347 | 2 | 12 |
| Raymond | 30 | 262 | 82 | 2 |
